# Macrostructural Alterations of Subcortical Grey Matter in Psychogenic Erectile Dysfunction

**DOI:** 10.1371/journal.pone.0039118

**Published:** 2012-06-18

**Authors:** Nicoletta Cera, Stefano Delli Pizzi, Ezio Domenico Di Pierro, Francesco Gambi, Armando Tartaro, Carlo Vicentini, Giuseppe Paradiso Galatioto, Gian Luca Romani, Antonio Ferretti

**Affiliations:** 1 Department of Neuroscience and Imaging, Institute for Advanced Biomedical Technologies (ITAB), University “G. d’Annunzio” of Chieti, Chieti, Italy; 2 Department of Health Sciences, University of L’Aquila, L’Aquila, Italy; 3 Hospital “G. Mazzini”, Teramo, Italy; Hangzhou Normal University, China

## Abstract

Psychogenic erectile dysfunction (ED) has been defined as the persistent inability to attain and maintain an erection sufficient to permit sexual performance. It shows a high incidence and prevalence among men, with a significant impact on the quality of life. Few neuroimaging studies have investigated the cerebral basis of erectile dysfunctions observing the role played by prefrontal, cingulate, and parietal cortices during erotic stimulation. In spite of the well-known involvement of subcortical regions such as hypothalamus and caudate nucleus in male sexual response, and the key role of nucleus accumbens in pleasure and reward, poor attention was paid to their role in male sexual dysfunction. In this study, we determined the presence of grey matter (GM) atrophy patterns in subcortical structures such as amygdala, hippocampus, nucleus accumbens, caudate nucleus, putamen, pallidum, thalamus, and hypothalamus in patients with psychogenic ED and healthy men. After Rigiscan evaluation, urological, general medical, metabolic and hormonal, psychological and psychiatric assessment, 17 outpatients with psychogenic ED and 25 healthy controls were recruited for structural MRI session. Significant GM atrophy of nucleus accumbens was observed bilaterally in patients with respect to controls. Shape analysis showed that this atrophy was located in the left medial-anterior and posterior portion of accumbens. Left nucleus accumbens volumes in patients correlated with low erectile functioning as measured by IIEF-5 (International Index of Erectile Function). In addition, a GM atrophy of left hypothalamus was also observed. Our results suggest that atrophy of nucleus accumbens plays an important role in psychogenic erectile dysfunction. We believe that this change can influence the motivation-related component of sexual behavior. Our findings help to elucidate a neural basis of psychogenic erectile dysfunction.

## Introduction

Psychogenic Erectile Dysfunction (ED) has been defined as the persistent inability to attain and maintain an erection sufficient to permit sexual performance. Moreover, psychogenic ED represents a disorder related to psychosocial health and has a significant impact on the quality of life of both sufferers and their partners. Epidemiological studies have shown a high prevalence and incidence of psychogenic ED among men.

In the last decade, numerous functional neuroimaging studies have focused on the brain regions that are evoked by sexually relevant stimuli, showing an involvement of different cortical and subcortical structures, such as cingulate cortex, insula caudate nucleus, putamen, thalamus, amygdala and hypothalamus [1]–[5]. These studies have permitted to disentangle the role played by several brain regions in different stages of visually driven sexual arousal. Indeed male sexual arousal has been conceived as a multidimensional experience involving cognitive, emotional and physiological components that relay on a widespread set of brain regions. Conversely, few neuroimaging studies have investigated the cerebral correlates of male sexual behavior dysfunction. These studies demonstrate that some brain regions, as, for example, the cingulate and frontal cortex, may have an inhibitory effect on the male sexual response [6]–[8]. However, numerous evidences [9]–[12] indicate the importance of subcortical structures in different stages of copulative behavior. Indeed, the hypothalamus plays a key role [4], [5] in the central control of penile erection. According to Ferretti and colleagues [4] the hypothalamus can be brain area that triggers the erectile response evoked by erotic clips.

Little is known about the role played by the remaining subcortical structures in male sexual behavior dysfunction. Among the deep grey matter (GM) regions, the nucleus accumbens plays a well recognized role in reward and pleasure circuits [13]–[16] and the caudate nucleus in the control of the overt behavioral response of sexual arousal [2].

The aim of this study is to investigate if psychogenic ED patients show macro-structural alterations of deep GM structures that are involved in the male sexual response, in pleasure and reward.

To test this hypothesis, structural MRI assessment of eight subcortical GM structures of the brain, such as the nucleus accumbens, amygdala, caudate, hippocampus, pallidum, putamen, thalamus and hypothalamus was performed on a study population of psychogenic ED patients and control subjects. If there are any differences between the two groups in some of these regions, our interest is to see the presence of a relationship between changes in specific brain area volumes and behavioral measures.

## Methods

### Ethics Statement

The study was approved by the ethics committee of University of Chieti (PROT 1806/09 COET) and conducted in accordance with the Helsinki Declaration. Protection of subject’s personal information and their intimacy were ensured by implementing the guideline suggested by Rosen and Beck [17]. The study design was explained in detail and written informed consent was obtained from all participants involved in our study.

### Study Design

97 patients who visited the outpatient’s clinic for sexual dysfunctions of the Division of Urology of the department of Health Sciences of University of L’Aquila between January 2009 and May 2010 were recruited for this study. Patients who visited the clinic complained of erectile dysfunction, whereas healthy subjects were recruited by means of a notice on a bulletin board at University of Chieti and Hospital of Teramo.

All participants were examined according to a standardized protocol including a general medical, urologic and andrologic examination, psychiatric and psychological screening and whole brain MRI.

### Subjects

Patients came to the outpatient’s clinic for sexual dysfunctions and difficulties experienced by the patients or notified by their partners. The patients were categorized as having psychogenic erectile dysfunction (generalized or situational types) or organic erectile dysfunction (vasculogenic, neurogenic, hormonal, metabolic, drug induced). The Urologic assessment was performed following current guidelines for diagnosis of erectile dysfunction [18].

The diagnostic evaluation of psychogenic erectile dysfunction (Generalized type) was performed by means of a physical examination with particular emphasis on the genitourinary, endocrine, vascular and neurological systems. Additionally, normal nocturnal and morning erections was evaluated by the Rigiscan device during three consecutive nights, while, normal penile hemodynamics was assessed using color Doppler Sonography. In total, 80 patients were excluded because most of them did not meet the criteria for enrollment in the experiment. Some of them were on antidepressants, or had hormonal deficits. However, all patients with psychogenic erectile dysfunctions were enrolled. The same clinical examinations were performed on control subjects. Normal nocturnal erection was also verified in the controls.

Seventeen right handed heterosexual outpatients with diagnosis of psychogenic erectile dysfunction (mean age ± SD=34.3±11; range 19–63) and twenty-five healthy right handed heterosexual men (mean age ± SD=33.4±10; range 21–67) were recruited for this study. Patients and healthy controls were matched not only in terms of ethnicity, age, education, but also in terms of nicotine use [19].

### Psychiatric and Psychological Assessment

All subjects underwent a 1-h medical history interview with a psychiatrist and took the Mini-International Neuropsychiatric Interview (M.I.N.I.) [20].

Erectile function, sexual arousability, psychophysical status, anxiety and personality were assessed using the following questionnaires: International Index of Erectile Function (IIEF) [21], Sexual Arousal Inventory (SAI) [22], SCL-90-R [23], State-Trait Anxiety Inventory (STAI) [24], and Behavioral Inhibition/Behavioral Activation Scale (BIS/BAS scale) [25], respectively.

### MRI Data Acquisition

Whole Brain MRI was performed by means a 3.0 T “Achieva” Philips whole body scanner (Philips Medical System, Best, The Netherlands), using a whole-body radiofrequency coil for signal excitation and an eight-channel head coil for signal reception.

A high resolution structural volume was acquired via a 3D fast field echo T_1_-weighted sequence. Acquisition parameters were as follows: voxel size 1 mm isotropic, TR/TE=8.1/3.7 ms; number of sections=160; no gap among sections; whole brain coverage; flip angle=8°, and SENSE factor=2.

### Data Analysis

Structural MRI data were analyzed using tool from Functional MRI of the Brain (FMRIB) Software Library [FLS, http://www.fmrib.ox.ac.uk/fsl/index.html] [26], [27] version 4.1. Before data processing, noise reduction of structural images was performed by using SUSAN algorithm [http://www.fmrib.ox.ac.uk/analysis/research/susan/].

### Volumes Measurement and Shape Analysis of Subcortical Structures

The FLIRT tool was used to perform affine alignment of the 3D T_1_ images on the MNI152 template (Montreal Neurological Institute) by means of affine transformations based on 12 degrees of freedom (i.e. three translations, three rotations, three scaling and three skews) [28], [29]. Subcortical grey matter (GM) structure segmentation and absolute volume estimation of amygdala, hippocampus, nucleus accumbens, caudate nucleus, putamen, pallidum and thalamus were performed using FIRST [30]. Successively, subcortical regions were visually checked for errors.

For each GM subcortical structure, FIRST outcomes provides a surface mesh (in MNI152 space) that is formed of a set of triangles. The apices of adjoining triangles are called vertices. Because the number of these vertices in each GM structure is fixed, corresponding vertices can be compared across individuals and between groups. Pathological alterations modify the vertex arbitrary orientation/position. In this way, the local shape changes was directly assessed by analysing vertex locations and by looking at the differences in mean vertex position between controls and patients groups. Group comparisons of vertices were carried out using F-statistics [30], [31]. Design matrix is a single regressor specifying group membership (zero for controls, ones for the patients).

### Estimation of Brain Tissue Volume

SIENAX [http://www.fmrib.ox.ac.uk/fsl/fast4/index.html#FastGui] was applied to estimate brain tissue volume. After brain and skull extraction, the original structural image of each subject was affine-registered to MNI 152 space as described in the previous section. Tissue-type segmentation [32] was performed to estimate the volumes of GM, white matter (WM), peripheral GM, ventricular CSF and total brain volume. Intracranial volume (ICV) was calculated by adding the volumes of cerebral spinal fluid, total GM and total WM together.

### ROI Voxel-based Morphometry (VBM) Analysis

According with the methods reported by literature [33], ROI-VBM analysis of hypothalamus was performed to assess the morphological changes occurring in ED patients than control subjects. ROI of right and left hypothalamus were manually drawn on the basis of MRI atlas [34].

Data were analyzed by using a VBM analysis [35], [36]. After brain-extraction using BET [37], tissue-type segmentation was carried out using FAST4 [32]. The resulting GM partial volume images were aligned to MNI152 standard space using the affine registration tool FLIRT [28], [29], followed by nonlinear registration using FNIRT [38], [39]. The resulting images were averaged to create a template, to which the native GM images were then non-linearly re-registered. For correcting local expansion or contraction, the registered partial volume images were then modulated by dividing by the Jacobian of the warp field. Finally, the patient and control groups were compared using voxel-wise statistic (5000 permutations) and the threshold-free cluster-enhancement option in the “randomize” permutation-testing tool in FSL [http://www.fmrib.ox.ac.uk/fsl/randomise/index.html]. To overcome the risk for false positives, the significance threshold for between-group differences was set at p<0.05 corrected for family-wise error (FWE). Correlation analysis with IIEF-5 and SAI was also performed.

### Statistical Analysis

Statistica® 6.0 was used for data analysis. ED patients and healthy controls were compared by means of an univariate analysis of variance (1-way ANOVA) for age, educational level, use of nicotine, ICV and volumes of deep grey structures separately. In order to minimize the likelihood of type I error, an overall multivariate analysis of variance (MANOVA) using single volumes of subcortical structures corrected for ICVs in each of the analyses as dependent variables. Then, 1-way ANOVAs (between groups) were run for each volume value. A level of significance of p<0.05 was used. Then, the possible relationship between behavioral measures and volume values is investigated. The mean volumes values and the behavioral measures, included in correlation analysis, are those that showed a significant between group differences. Correlation analysis was performed by means of Spearman’s rho coefficient, for the two groups separately, corrected for multiple comparisons (p<0.05).

## Results

Demographic features for the two groups are shown in **Table 1**.

**Table 1:** Demographics characteristics.

Demographics				
	Patients	Controls	F	*p level*
**N**	17	**25**		
**Age in Years (M, SD)**	34,3±11	**33,4±10**	**0,082**	***0,77***
**Education in Years (M, SD)**	14,3±4	**14,8±3**	**0,019**	***0,89***
**ICV in mm^3^ (M, SD)**	1532415,6±67771,8	**1514682±58693,9**	**0,82**	***0,37***
**Nicotine Cigarettes/day (M, SD)**	5,7±7,5	**4,6±5,6**	**0,304**	***0,58***

M=mean; SD=standard deviation; ICV=Intra Cranial Volume.

ED patients and healthy controls did not differ significantly for age, educational level, consumption of nicotine and ICV (Intra Cranial Volume in mm^3^), grey and white matter volumes and total brain volume.

Significant between group difference was found for total score of IIEF-5 with higher values in the control group than patient group (F_(1,40)_=79; p<0.001), and for total score of SAI with a F_(1,40)_=13 and p<0.001). Particularly, for the subscale “Excitation” of the SAI healthy controls showed a significantly higher mean score than ED patients (F_(1,40)_=22.3; p<0.001). Neither anxiety, as measured by STAI, nor personality, as measured by BIS/BAS scale, showed significant between group differences. A significant difference was seen for subscale “Fun Seeking” of the BIS/BAS scale with a higher mean score for controls than patients (F_(1,40)_=5.2; p<0.05).

**Figure 1:**
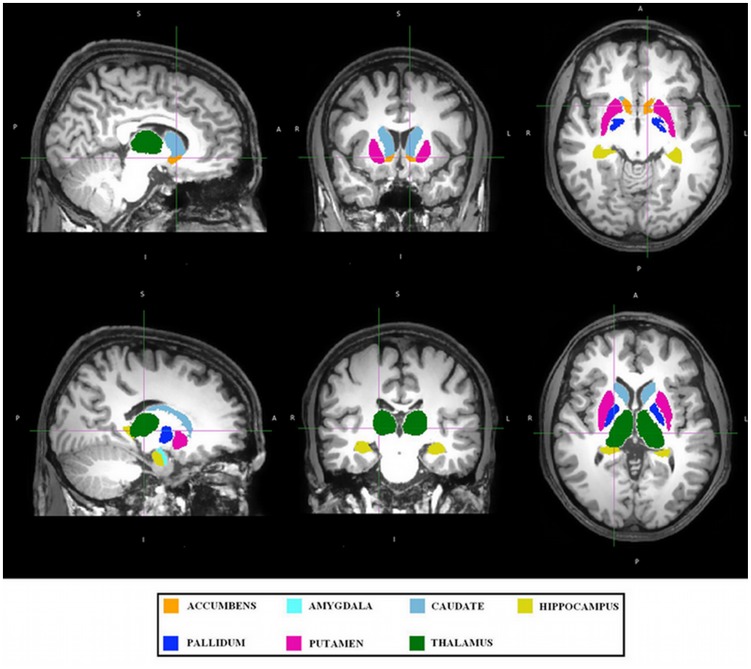
Segmentation of the deep grey matter structures. Images are over imposed on MNI template.

In each subject 7 subcortical structures (thalamus, hippocampus, caudate, putamen, pallidum, amygdala, and accumbens) were segmented and their volumes measured with FIRST tool (Fig.1). Table 2 reports the mean volumes (M) and standard deviation (SD) of the above mentioned regions in cubic millimeters for ED patients and control groups. Table 3 shows the mean volumes of the subcortical structures in patient and control groups for the two brain hemispheres separately. A MANOVA indicated the presence of between group differences in the subcortical areas (Wilks λ=0.58; F=3,45; p=0.006). Then, a series of follow up one-way ANOVAs revealed a significant decrease in volume of the nucleus accumbens in ED patients compared to controls (F_(1,40)_=11,5; p=0.001).

**Table 2:** Mean volumes of subcortical structures in cubic millimeters for Psychogenic ED patient and healthy control groups.

Mean volumes (mm^3^)				
	Patients		Controls	
	M	SD	M	SD
**Thalamus**	9813,11	1067,4	**10151,9**	**605,56**
**Caudate Nucleus**	4640,14	665,28	**4955,18**	**444,87**
**Putamen**	6052,41	936,32	**6379,88**	**590,35**
**Pallidum**	2191,88	278,52	**2236,82**	**199,85**
**Hippocampus**	4842,73	779,47	**5146,38**	**507,94**
**Amygdala**	1585,64	241,35	**1518,48**	**228,68**
**Nucleus Accumbens**	565,58*	135,1	**685,58**	**93,79**

A MANOVA was applied for estimating between group differences(p<0.05) and consecutively follow up 1-way ANOVAs on the volume of each subcortical area.

* p<0.001, with a reduction in volumes for the patient group. M=mean; SD=standard deviation.

**Table 3:** Mean volumes of subcortical structures in cubic millimeters for Psychogenic ED patient and healthy control groups and for the two brain hemispheres separately.

Mean volumes (mm^3^)								
	Patients				Controls			
	L		R		L		R	
	M	SD	M	SD	M	SD	M	SD
**Thalamus**	10071,53	1027,08	9554,71	1163,50	**10444,52**	**558,5**	**9859,28**	**709,71**
**Caudate Nucleus**	4704,71*	677,12	4575,59	669,30	**5060,6**	**435,51**	**4849,76**	**473,29**
**Putamen**	6029,24	912,40	6075,59	1016,70	**6296,24**	**536,49**	**6463,52**	**710,3**
**Pallidum**	2186,00	308,41	2197,76	274,14	**2265,52**	**194,14**	**2208,12**	**236,13**
**Hippocampus**	4853,41	781,81	4832,06	1023,05	**5089,24**	**597,14**	**5203,52**	**648,07**
**Amygdala**	1619,53	290,13	1551,76	262,72	**1588,92**	**234,19**	**1448,04**	**320,02**
**Nucleus Accumbens**	566,59*	185,02	564,59*	106,54	**713,56**	**120,36**	**657,6**	**91,15**

A MANOVA was applied for estimating between group differences(p<0.05) and consecutively follow –up 1-way ANOVAs on the volume of each subcortical area.

* p<0.005, with a reduction in volumes for the ED patient group. M=mean; SD=standard deviation.

An additional MANOVA, performed on the values of volumes of the left and right subcortical regions, revealed a significant differences between ED patients and controls (Wilks λ=0.48; F=2,09; p=0.04). Consequently, follow–up one –way ANOVAs showed significant decreased volumes of left and right nucleus accumbens in ED patients with respect to healthy controls (F_(1,40)_=9.76; p=0.003; F_(1,40)_=9.19; p=0.004 respectively).

**Figure 2:**
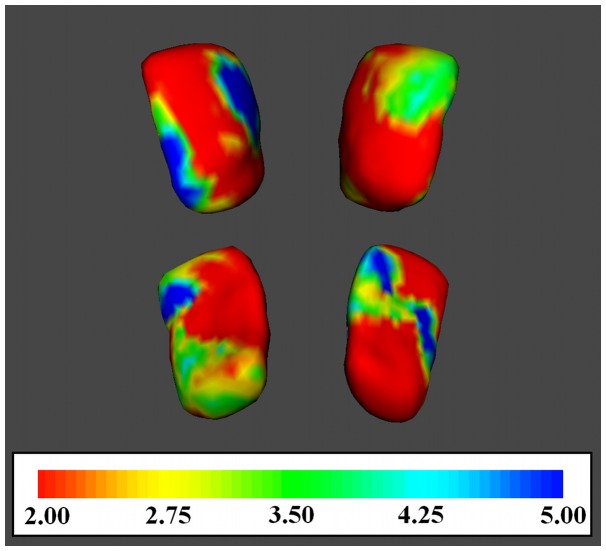
Vertex-wise comparison of the nucleus accumbens between healthy controls and Psychogenic ED patients. For vertex-wise shape analysis F-statistics have degrees of freedom of 1,40 giving p=0.165 (F=2), p=0.105 (F=2.75), p=0.086 (F=3.50), p=0.045 (F=4.25), p=0.030 (F=5). Family-wise error rate is controlled. Images are oriented according to neurological convention (the right hemisphere of the brain corresponds to the right side of the image).

The results of shape analysis performed on the nucleus accumbens are shown in Figure 2.

The comparison of vertex location between the two groups showed significant regional atrophy in ED patients in correspondence to the left medial-anterior and, bilaterally, to the posterior portion of the nucleus accumbens.

**Figure 3:**
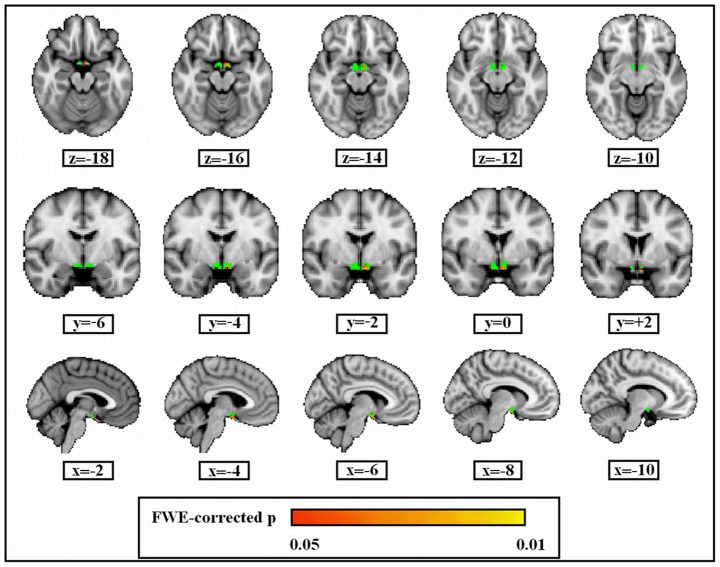
Grey matter volume loss of left lateral hypothalamus in ED patients than healthy subjects. Green colour describes the extent of the hypothalamus ROI. The significant voxels (family-wise error rate is controlled) are projected onto a Montreal Neurological Institute (MNI152) template and identified by colours ranging from red to yellow (the colored bar represents the p-value). All images are oriented according to radiological convention (the left hemisphere of the brain corresponds to the right side of the image).

As reported in Figure 3, ROI-VBM analysis showed a GM atrophy in the left hypothalamus (p<0.05, the FWE rate is controlled). Specifically, GM loss was found in the supraoptic nucleus of the anterior hypothalamic area (*x, y, z coordinates: −6, −2, −16,* p=0.01corrected), the ventromedial nucleus of the hypothalamus (*x, y, z coordinates: −4, −4, −16*, p=0.02 corrected), and the medial preoptic nucleus (*x, y, z coordinates:−4, 0, −16,* p=0.03 corrected).

The correlation analysis was performed between the behavioral measures (IIEF and SAI) and FIRST and ROI-VBM outcomes. Positive correlations were observed between IIEF mean scores and left nucleus accumbens in the patient group (rho=0,6; p<0.05, corrected for multiple comparison) and between SAI total score and the left hypothalamus (p=0.01, the FWE rate is uncontrolled).

## Discussion

Our study explored the patterns of subcortical region atrophy in male psychogenic erectile dysfunction. Structural MRI analysis revealed a significant GM atrophy of both left and right nucleus accumbens and left hypothalamus in patients diagnosed with psychogenic ED dysfunction of the generalized type with respect to healthy controls. These macro-structural alterations were independent of age, nicotine consumption, educational levels and intracranial volume. Further, GM atrophy of the left nucleus accumbens showed a positive correlation with poor erectile functioning in patients, as measured by International Index of Erectile Function (IIEF). Moreover, the GM volume loss in the left hypothalamic regions were related to the Sexual Arousability Inventory (SAI) scores which represents another measure of sexual behavior. Both these subcortical regions participate in many neural pathways with functions related to autonomic control and emotions.

Based on our results, the principal finding of the present study is represented by GM atrophy observed in the nucleus accumbens of the patient group. The role played by the nucleus accumbens in male sexual behavior was supported by physiological evidence in the male rat [40] and by functional neuroimaging studies in healthy men during visual erotic stimulation [2]. The release of dopamine in the nucleus accumbens drives the mesolimbic system that is involved in behavioral activation in response to sensory cues signaling the presence of incentives or reinforcers [41]. This is supported by physiological evidence linking the dopaminergic activity in the NAcc to sexual appetite behavior in male rat [40], [41]. Indeed an increased level of dopamine in the nucleus accumbens of the male rat is observed when a female rat was introduced to him. This increase was reduced during the post copulatory refractory period.

In the light of this, activity in the nucleus accumbens was associated with regulation of emotional responses. The human nucleus accumbens seems to be selectively reactive to pleasant pictures stimuli rather than salience [42]. According to Redoutè and colleagues [2] the nucleus accumbens is likely to participate in the motivational component of male sexual arousal. The human nucleus accumbens is activated during erection evoked by visual erotic stimulation [1], [2].

Moreover, our results on the shape differences seems to be in line with the motivational hypothesis, given that the observed atrophy involves principally the shell of the nucleus accumbens. Shell represents a region that appeared particularly related to motivation and appetitive behaviors [43], [44]. In the male rat the selective electrophysiological inactivation of the shell, but not the core of the nucleus accumbens, seems to increase responding to the non-reward cue [45].

Our findings are in line with previous animal evidences that have observed how the release of dopamine from the nucleus accumbens and the medial preoptic area of the hypothalamus seems to positively regulate the motivational phase of copulatory behavior.

In this way, the hypothalamus represents an essential region for stimulating erectile function [3], [4]. We found a decrease in grey matter volume of the lateral hypothalamus in patients with psychogenic erectile dysfunction. These changes in grey matter volume were observed in the area of the supraoptic nucleus of the anterior hypothalamic area, medial preoptic and ventromedial nucleus. According to a series of experimental evidences, the medial preoptic area and anterior portion of the hypothalamus play a crucial role in the control of male sexual behavior in every mammalian species [46]. Specifically, bilateral lesions of these hypothalamic regions irreversibly abolish male sexual drive in rats [47], [48]. Taken together, these studies show that bilateral lesions of medial preoptic nucleus and the anterior hypothalamus impair sexual motivation in rats [40], [47], [49]. Moreover, increased activity during sexual motivation, hunger and aggression has been seen [50]. Georgiadis and colleagues [5] showed how different subsections of the hypothalamus are selectively related to different stages of erection in healthy men. Indeed, the lateral hypothalamus correlated with penile circumference and seems to be associated with aroused states. Functional neuroimaging studies have shown that other subcortical structures, such as the hippocampus, the amygdale and thalamus presented high activity in relation to visual erotic stimulation and to specific stages of penile erection [4]. According to our results, there were no changes in volume of these deep grey structures in the patient group.

It is noteworthy that this study has some limitations. Since the FIRST tool does not include hypothalamus segmentation, the ROI-VMB analysis represents the most reliable solution for automatically assessing the macro-structural changes in the hypothalamus. But this approach was not originally designed for the analysis of sub-cortical structures, being prone to artefact generation in the subcortical GM. VMB is based on locally-averaged GM segmentations and is therefore sensitive to the inaccuracies of tissue-type classification and arbitrary smoothing extents [30], [51]–[53]. For this reason the interpretation of ROI-VBM findings require some caution.

### Conclusion

In spite of the growing interest of cerebral correlates in sexual behavior, male sexual dysfunctions have received poor attention. Our findings emphasize the presence of macro-structural changes in GM of two subcortical regions, the nucleus accumbens and the hypothalamus, that seem to play an important role in the motivational aspects of male sexual behavior. Our findings highlight the importance of the motivational component of sexual behavior to permit satisfactory sexual performance in healthy men. Moreover, it may be plausible that inhibition of the sexual response in patients affected with psychogenic erectile dysfunction may act on this component. The alterations of subcortical structures taken together with previous functional neuroimaging evidences shed new light on the complex phenomenon of sexual dysfunction in men.

Furthermore, these results can help to develop new therapies for the future and to test the effect of those currently in use.
